# Probing Rigidity and Fluidity in the Interfacial Region of Lipid Bilayers with a Novel IR Probe

**DOI:** 10.1021/acs.jpcb.5c06183

**Published:** 2025-10-22

**Authors:** Md Muhaiminul Islam, Sithara U. Nawagamuwage, Cameron A. Dennis, Igor V. Rubtsov

**Affiliations:** Department of Chemistry, 5783 Tulane University, New Orleans, Louisiana 70118, United States

## Abstract

We report on development of a vibrational probe (N_3_–C_12_H_25_, az12) suitable for reporting on the rigidity and fluidity of the interfacial region in lipid bilayers. Such probe can help assess critical biological functions of cell membranes, including membrane permeability. We demonstrated a high sensitivity of the az12 probe to the rigidity of the interfacial region, manifested in the sensitivity of the width of the N_3_ moiety asymmetric stretching mode absorption peak. The structural and dynamic changes associated with the gel–liquid-crystal phase transition (Lβ-Lα) for three different lipids dipalmitoylphosphatidylcholine (DPPC), dipalmitoylphosphatidylglycerol (DPPG), and egg sphingomyelin (SM) were studied using FTIR and two-dimensional infrared (2DIR) spectroscopies. In addition to the new az12 probe targeting the interfacial region, the N_3_-(CH_2_)_11_–CN probe (az11CN), recently developed to examine the hydrophobic region of bilayers, was also used, showing characteristic phase transitions for each bilayer at their characteristic phase transition temperatures. An order parameter, *S*_N3_, corrected for the difference in the polarities of the interfacial and hydrophobic regions, was constructed. It shows how the overall rigidity of the bilayer is divided between the interfacial and hydrophobic regions, emphasizing their correlations. 2DIR spectral diffusion data were acquired for az12 and az11CN probes in the bilayer at various temperatures, reporting on inhomogeneous and homogeneous line width contributions (rigidity) and correlation times (fluidity) for the interfacial and hydrophobic regions. The spectral diffusion data for the interfacial region for all three bilayer types show large static inhomogeneous contributions, which are absent in the hydrophobic region data. The spectral diffusion data revealed differences for different bilayers, most apparent for the interfacial region. A greater increase in a homogeneous line width for SM with temperature, compared to that for DPPC and DPPG, could be linked to an increase in water permeability to the interfacial region of SM at higher temperatures. We found that the az12 probe constitutes a powerful reporter to measure rigidity (exerted angular constraints) and fluidity (time responses of the environment) in the interfacial region of a bilayer.

## Graphical abstract

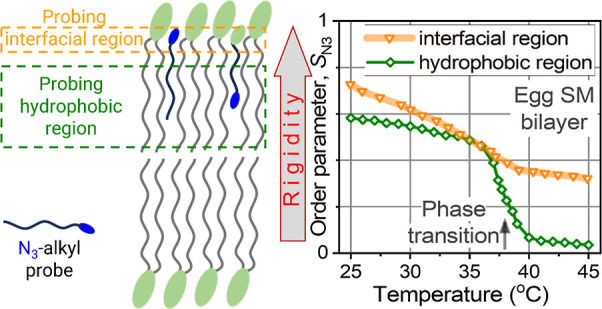

## 1 Introduction

A cell membrane not only defines the boundary of the cell but also performs critical physiological functions, such as enzymatic reactions, regulation of the transport of nutrients into the cell, expelling toxic substances, and supporting a wide range of other biological activities.^1^ The lipid bilayer acts as a host matrix for various membrane components, playing an essential role in regulating the function of integral membrane proteins.^2–5^ For example, the changes in membrane potential affect the activity of ion channel proteins.^2,6^ Properties like fluidity and thickness are essential in determining the membrane’s functional capabilities,^7,8^ and these properties are interdependent, making their study particularly challenging as interactions of membrane components span multiple length and time scales.^9–12^ The development of a robust method for characterizing membrane properties in naturally occurring cell membranes is crucial for advancing our understanding of cell membrane biochemistry.

A typical cell membrane consists of hundreds of lipids and proteins, creating a laterally heterogeneous structure with dynamic nanoscale domains referred to as lipid rafts.^13–23^ Lipid rafts play a vital role in membrane trafficking, sorting, signal transduction, and cell polarization, among other cellular processes.^24–26^ However, the nanoscopic scale of these rafts poses challenges for conventional microscopy techniques due to the diffraction limit.^27–30^ Various biophysical techniques have been used to investigate the properties of plasma membranes, yet their findings often conflict. Some studies suggest that cholesterol has little influence on the stiffness of unsaturated membranes, while others report a similar effect of cholesterol on the rigidity to that observed in saturated lipids.^31–35^ Other properties like phase transition^36–39^ and elasticity^40,41^ were found to vary in literature. One potential reason for the discrepancies in literature is the inherently high heterogeneity of the bilayer. Nonperturbing and site-specific labeling within cellular membrane domains has long been, and continues to be, one of the foremost challenges in the field. The experimental approach developed in this study has the potential to address nanoscale domain properties in cell membranes and provide in-depth information on the role played by lipid rafts in cellular processes.

Conformational motion in cell membranes and their components spans a wide range of time scales with many of these dynamics intricately interconnected.^42,43^ Since the structure and function of membrane proteins are influenced by bilayer dynamics, understanding the lipid bilayer’s behavior is critical.^44^ While techniques like NMR, ESR, and X-ray/neutron scattering can probe fast time scales, directly observing local structural rigidity and dynamic changes (fluidity) in bilayers remains an experimental challenge.^45–49^ Vibrational spectroscopy offers a promising solution, providing a nonperturbative and nondestructive method to probe complex biomolecular systems. However, despite the critical importance of ultrafast studies on biological membranes, the application of vibrational spectroscopy to cell membranes has been largely constrained by the limited availability of suitable vibrational probes.

The Rubtsov group has recently developed a vibrational spectroscopic approach for measuring the rigidity and fluidity in the hydrophobic region of a lipid bilayer utilizing probe compounds containing an azido group tethered to a linear alkyl chain terminated with a more polar end group, cyanide (CN) or carboxylic acid (AC).^50,51^ Such probe compounds, az*n*CN and az*n*AC, where *n* is the number of carbon atoms in the alkyl chain, were shown to orient themselves in the bilayer such that the more polar group resided near the bilayer’s headgroup and the azido group was positioned in the hydrophobic region at depths determined by the length of the alkyl chain, *n*. While the azido moiety generally favors regions of intermediate polarity, such as the interfacial region, its attachment to an alkyl chain terminated with a more polar group forces it into the hydrophobic interior of the bilayer. An asymmetric stretching mode of the azido group, ν_N3_, was found to be a unique reporter, capable of detecting sensitively variations in bilayer rigidity and fluidity.

In this study, we demonstrate that an azido moiety connected to an alkyl chain with no additional polar group resides near the interfacial region of a lipid bilayer, providing insights into the polarity, rigidity, and dynamics (fluidity) of this region. We employed an az12 compound (N_3_–C_12_H_25_, Figure 1) to probe the interfacial regions of three bilayers made of saturated lipids with distinctly different head groups and interfacial structures and used the previously established az11CN label to investigate the effects of different head groups in the hydrophobic region. The lipids were selected to include a pair with similar head groups but different interfacial motifs (DPPC vs SM) while another pair features similar interfacial motifs but different head groups (DPPC vs DPPG). Note that the lipid selection considered the biological significance of these lipids, ensuring relevance for broader studies on membrane systems, such as lipid rafts and bacterial cell membranes.^52–54^

**Figure 1:**
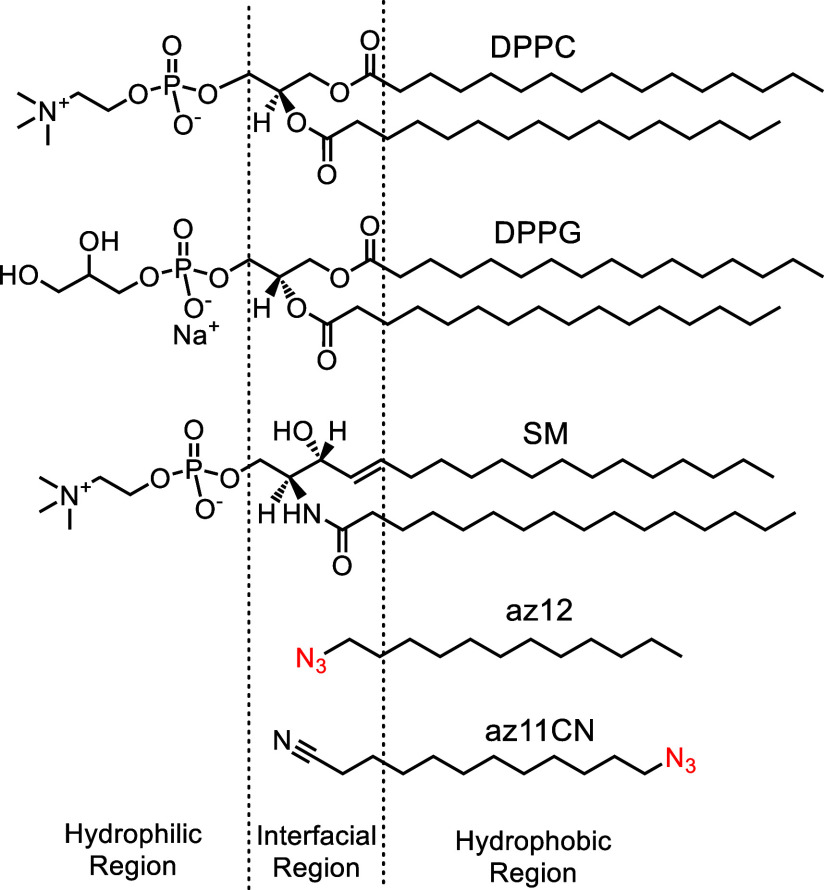
Structures of the lipids, DPPC, DPPG and SM, and probe compounds 12az and az11CN.

It is worth noting that the ester carbonyl group and the amide group in SM present intrinsic vibrational probes in the cell membrane’s interfacial region and have been studied extensively as such.^55–57^ While the vibrational mode of a carbonyl group can serve as a reporter on the medium polarity, it does not show direct sensitivity to the rigidity of its surroundings, as the azido group does. Also, in complex membrane environments, carbonyl vibrations are challenging to interpret due to the presence of two CO groups per lipid with varied vibrational lifetimes and hydrogen bonding conditions. Vibrational coupling and energy transfer dynamics within the lipid and between different lipids complicate the analysis.^56^ Moreover, discrepancies exist in the literature regarding the origin of those two peaks observed in the ester region. Some studies attribute this phenomenon to variations in hydrogen bonding configurations, while others suggest that it arises from the spectroscopic differences between the sn-1 and sn-2 carbonyl groups.^56,58–60^

Importantly, the azido group is particularly well-suited for assessing environmental rigidity due to its intrinsic inhomogeneity, stemming from a wide distribution of allowed CNN angles (θ).^51^ Utilizing a probe that can be strategically positioned at various locations within the bilayer is essential, as it enables a comparative analysis that deepens our understanding of the structural and dynamic properties across different bilayer regions. Having vibrational frequency in the essentially transparent region (∼2100 cm^–1^) makes the use of the N_3_-alkyl probes convenient. In this study we will show that the azido group can probe both hydrophobic and interfacial regions, facilitating direct comparisons between these areas.

## 2 Experimental Details

1,2-Dipalmitoyl-*sn*-glycero-3-phosphocholine (DPPC), 1,2-dipalmitoyl-*sn*-glycero-3-phospho-rac-(1-glycerol) sodium salt (DPPG), and egg sphingomyelin (SM) were purchased from Avanti Polar Lipids. The probe compound, 1-azidododecane (az12) was obtained from Synthonix, while 12-azidododecanenitrile (az11CN) was synthesized in-house.^51^ The molecular structures of the lipids and the probe compounds are illustrated in Figure 1, along with a depiction of different regions of the lipid bilayer. Additionally, Figure 1 highlights the orientation of the labels and the relative position of the azido group within the bilayer. All the experiments were done in planar multilamellar bilayer (MLBL) prepared by isopotential spin-dry ultracentrifugation method with 1:10 guest/lipid molar ratio (see Section S3).^61^

The sample obtained from the ultracentrifuge was sealed between two 1 mm thick CaF_2_ windows. To maintain precise temperature control, the sample was enclosed within an insulation jacket, and the temperature was monitored using a thermocouple, ensuring consistency within ±0.2 °C throughout the FTIR and 2DIR measurements. The 2DIR spectra were recorded using a fully automated, dual-frequency, three-pulse photon echo spectrometer, as detailed in previous reports and Section S2 of Supporting Information.^62,63^ All the measurements were repeated three or more times.

## 3 Results and Discussion

### 3.1 Temperature Dependent FTIR Measurements

The central frequency of the azido moiety peak, ν_N_3__, shows high sensitivity to the polarity of the environment featuring higher peak frequency in more polar media.^50,64^ Therefore, it is not surprising that the az11CN probe, with the azido moiety located in the middle of a single leaflet of the bilayer, exhibits the same central frequency (∼2095.5 cm^–1^) regardless of the lipid nature, indicating similar polarities in this region (Figure 2B). On the contrary, the az12 probe shows different central frequencies for different lipid types (∼2097 cm^–1^ in DPPC and DPPG and ∼2098 cm^–1^ in SM), with the values distinct from those for az11CN (Figure 2A inset). The higher frequencies for az12 in all the bilayers, compared to az11CN, indicate that its N_3_ group is located in the region of greater polarity.

**Figure 2:**
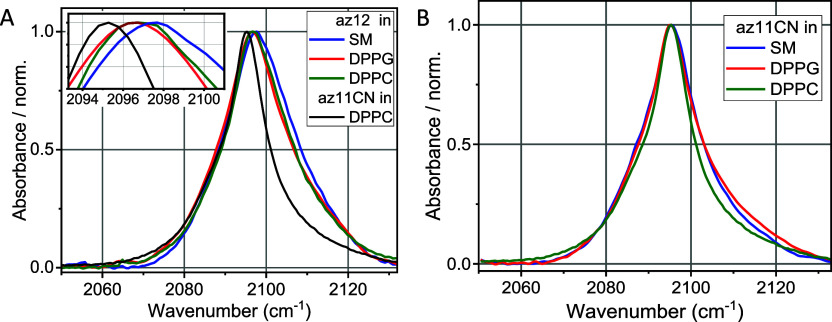
Water subtracted normalized FTIR spectra of ν_N3_ in bilayers made of different lipids, DPPC, DPPG, and SM with (A) az12 and (B) az11CN probes at *T* = 25 °C. Panel (A) also shows the ν_N3_ spectrum of az11CN in DPPC bilayer for the central frequency comparison, emphasized in the inset.

The lipids selected for the study are convenient for performing pairwise comparison of the az12 probe behavior as the DPPC and DPPG lipids feature the same interfacial moieties but different head groups, while DPPC and SM feature the same head groups but different interfacial structures. The similarity of the az12 peak frequencies in the DPPC and DPPG bilayers suggests that the N_3_ group is not exposed to the headgroup region but is located in the interfacial region. The difference in ν_N_3__ of az12 in DPPC and SM featuring the same head groups (Figure 2A inset) also indicates that the N_3_ moiety is not exposed to the head groups but located in the interfacial region of lipid backbones. The higher central frequency of az12 in SM bilayer indicates that the local polarity of the region around the azido group is higher than that of DPPC and DPPG. Indeed, the polarity of an amide group of SM is higher than that of an ester,^65^ which further supports the conclusion that the azido group is located in the interfacial region of the bilayer.

The width of the ν_N3_ absorption peak in alkyl azido groups is governed primarily by two factors. First is the polarity of the surrounding environment, which affects the bandwidth in a manner consistent with affecting other vibrational modes. Second is the range of accessible CNN bond angles (θ), determined by the local rigidity around the azido group. In our earlier work we showed that in the hydrophobic core of lipid bilayers—where polarity is essentially constant—the ν_N3_ bandwidth can vary by more than 12 cm^–1^ solely due to differences in the rigidity. This sensitivity arises from the softness of the CNN angle, resulting in a broad distribution of θ angles. The presence of multiple, N_3_-localized Fermi resonances lead to a broad ν_N3_ peak, featuring internal inhomogeneity that is larger than solvent induced inhomogeneity in solvent of medium polarity.^51^ When the angular θ distribution is restricted by the environment, as in the gel phase of a lipid bilayer hydrophobic region, the ν_N3_ peak width becomes narrow. Consequently, we used the degree of CNN angular restriction as a quantitative probe for assessing the local rigidity of the molecular environment.^51^ Furthermore, a time evolution of ν_N3_ can be measured via 2DIR spectral diffusion experiments, characterizing the local fluidity of the bilayer (Section 3.2). While in the hydrophobic interior of a bilayer, the ν_N3_ peak width depends solely on the rigidity. In the interfacial region the polarity also affects the ν_N3_ peak width, complicating the correlation of the ν_N3_ peak width with the rigidity. Careful analysis is therefore required for az12 to disentangle the polarity and rigidity contributions.

Figure 3A shows the changes in the peak width (σ_N3_, fwhm) with temperature for both probes, az12 and az11CN, in the three lipid bilayers. As expected, the ν_N3_ width of the az11CN probe shows strong sensitivity to the rigidity in the hydrophobic region of the bilayer,^51^ featuring small peak widths in the gel phase at temperatures (*T*) below the phase transition temperature (*T*_ph_) and much larger widths in the fluid (liquid crystalline) phase at *T* > *T*_ph_. Notice an abrupt increase in the width at *T*_ph_, which occurs at ca. 41 °C for DPPC and DPPG and at ca. 38° for SM. Such changes with temperature in the azido bandwidth were not observed in either polar or nonpolar solvents.^51^ The data for az11CN reflect predominantly the change in rigidity of the bilayer interior as the polarity of the bilayer interior deep in the saturated portion of the lipid is not changing with temperature, confirmed by a constant peak frequency, ν_N3_ (Figure 2B). Nevertheless, the rigidity of the bilayer interior at room temperature, reflected by σ_N3_, is different for different lipids, following the trend SM > DPPG > DPPC (Figure 3).

**Figure 3:**
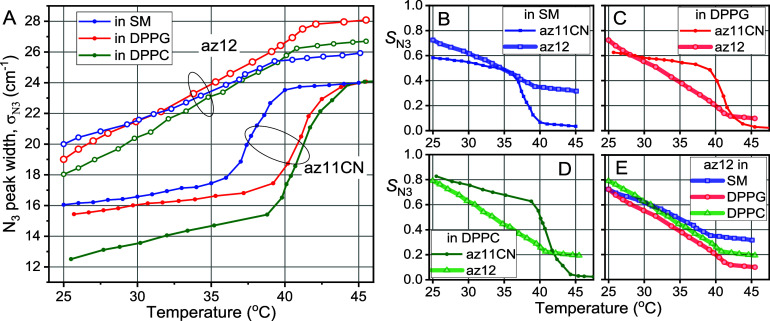
Temperature dependences of (A) the N_3_ peak width and (B–E) the order parameter, *S*_N3_, for az12 and az11CN in three lipid bilayers indicated. The error bars for individual points in panel A were at ± 0.3 cm^–1^.

The difference in the σ_N3_ width for az11CN in DPPC and DPPG indicates that the rigidity of the hydrophobic region is dependent on the nature of the lipid headgroup, which affects the area per lipid (APL). Indeed, larger APL values were observed in PG bilayers compared to PC.^66^ Electrostatic repulsion between negatively charged PG headgroups was linked to larger APL values in DPPG, leading to reduces molecular packing in the hydrophobic region of the bilayer.^67,68^

The difference in the σ_N3_ width for az11CN in DPPC and SM, featuring the same headgroups, indicates that the rigidity deep inside the bilayer is affected significantly by the structure of the interfacial region. The presence of a double bond in one of the SM lipid chains and the presence of an amide group in its interfacial region is likely the cause of the lower rigidity of the SM hydrophobic region at room temperatures. Interestingly, σ_N3_ of az11CN at *T* > *T*_ph_ is the same for all the lipids, indicating similar rigidity in the hydrophobic region in all the bilayers in the liquid-crystal state.

The peak widths of az12 for all three lipid types are larger than those for az11CN for all temperatures (Figure 3), significantly affected by a higher polarity in the interfacial region, observed by a ν_N3_ peak blue shift of az12 (Figure 2A). However, the polarity of the interfacial region is changing only slightly with temperature,^69^ so the strong temperature dependences observed for az12 must come from the changes in rigidity of the interfacial region, vide infra. Note also that the width of ν_N3_ in the interfacial region is found to be significantly smaller than that in the solvent of similar polarity (32 cm^–1^ in ethyl acetate). Therefore, steric motion hindrance in the interfacial region, the rigidity, affects significantly the peak width of ν_N3_. Electrostatic interactions in the interfacial region can induce such motion hindrance. The capability of the az12 probe to detect such interactions in the interfacial region rather sensitively is exciting.

To compare rigidities in different environments, the influence of the polarity on σ_N3_ have to be eliminated. We constructed an order parameter, *S*_N3_,^51^ that focuses on the rigidities of both environments, hydrophobic and interfacial. It is computed using eq 1, where σ_N3_^max^ and σ_N3_^min^ are the ν_N3_ widths observed in the environments that provide no structural constraints (low rigidity) and the largest structural constraints (high rigidity), respectively.

1 $$S_{N_{3}}=\left(\sigma_{N_{3}}^{\max}-\sigma_{N_{3}}\right)/\left(\sigma_{N_{3}}^{\max}-\sigma_{N_{3}}^{\min}\right)$$

The σ_N3_^max^ values were taken as σ_N3_ in the solvents of polarity matching that of the environment, hexadecane for az11CN (24.5 cm^–1^, Figure S1d) and ethyl acetate for az12 (29.5 cm^–1^, Figure S1d). The influence of the polarity on σ_N3_ is estimated as 5 cm^–1^, as the difference in width in the two solvents. The σ_N3_^min^ value for az11CN was taken as an extrapolation of σ_N3_ to smaller temperatures for the most rigid bilayer, DPPC, estimated at 10 cm^–1^. For az12, the σ_N3_^min^ value has increased by 5 cm^–1^ to account for the larger polarity of the interfacial environment, leading to σ_N3_^min^ = 15 cm^–1^. Such selection of σ_N3_^max^ and σ_N3_^min^ values effectively eliminates the influence of higher polarity of the interfacial region, making the order parameter for az12 to report solely on the rigidity of the environment (as if the environment is nonpolar). We believe that the implemented strategy, while approximate, represents adequately the main tendencies of the order parameter for both probes, az11CN and az12. Note that a systematic mapping of viscosity and specific-interaction effects on ν_N3_ peak width is an important direction for future studies.

The temperature traces of the order parameter obtained for az12 and az11CN are shown in Figure 3B–D. For DPPG and DPPC bilayers (Figure 3C,D), the order parameter for the hydrophobic region (az11CN) is generally larger than that for the interfacial region (az12), except for the high temperatures exceeding *T*_ph_. The situation is different for SM for which the interfacial region is more rigid than the hydrophobic region for most temperatures (Figure 3B). This result is caused by a smaller order in the hydrophobic region of SM, as discussed above, and by a higher order in its interfacial region. The latter is supported by the presence of both hydrogen bond donor and acceptor groups in SM interfacial region, resulting in intricate hydrogen bonding network, which makes the interfacial region stiffer. Apparent from the data, this network involves stronger interactions in its interfacial region,^70^ which remain mostly intact at temperatures above *T*_ph_, despite an increase in APL associated with the phase transition. These results agree with general recognition of SM for its higher overall rigidity,^17^ offering details on how this rigidity in distributed between the hydrophobic and interfacial regions (Figure 3B).

Figure 3E shows the order parameter data for the interfacial region for an easier comparison of the three bilayer types. The *S*_N3_(*T*) traces for DPPG and DPPC follow each other closely with the DPPC bilayer showing a slightly higher order parameter (+0.07). The comparison of these two bilayers identifies how the headgroup affects the interfacial region as the two lipids have identical interfacial regions. Similar to the trend observed in the hydrophobic region, we attribute this difference to the negative charge on the DPPG headgroup, which induce larger APL values in DPPG.^71^

The trace for SM is less tilted than those for DPPG and DPPC, which results in a smaller order parameter for SM at low temperatures and larger order parameter at high temperatures (Figure 3E). The weaker temperature dependence for *S*_N3_ of SM indicates stronger interactions in its interfacial region not affected as much by temperature.

Interestingly, in all three bilayer types, the order parameter of az12 decreases steadily with temperature until *T*_ph_ is reached, at which point the slope (d*S*_N3_/d*T*) decreases by ca. 10-fold. Before comparing different lipids, we discuss the steady rise of the width of az12 with temperature. Naturally, the traces do not show a decrease in *S*_N3_ at *T*_ph_ as this temperature is associated with elimination of crystallinity in the hydrophobic region of the bilayer while the azido group of as12 is located outside of the hydrophobic region. Nevertheless, the d*S*_N3_/d*T* derivative shows a strong sensitivity to temperature with the slope of ca. −0.03 °C^1–^ (or ca. 0.53 cm^–1^/°C for dσ_N3_/d*T*) for *T* < *T*_ph_ for DPPC and DPPG. We associate the changes in *S*_N3_ with continuous reduction in lipid chain crystallinity.^72^ Recent experiments targeting bilayer rigidity at different depths have found that the highest crystallinity, associated with the smallest ν_N3_ peak width at temperatures just below *T*_ph_ and the largest jump in the width at *T*_ph_, is observed at the “center” of the lipid chains, probed with az11CN and az10AC probes.^50,51^ It was shown that the chain crystallinity away from the center is gradually reduces. For example, the jump in the N_3_ width at *T*_ph_, detected with az8AC and az13AC, is only ca. 2.5 cm^–1^, while it is ca. 9 cm^–1^ probed at the center of a leaflet. Therefore, at temperatures below *T*_ph_, the crystallinity is gradually reduced as temperature increases, due to gradual melting of lipid microcrystals at lipid chain ends. This gradual melting results in softening the bilayer, a potential increase in the number of CH_2_ gauche conformations, and a decrease in the bilayer thickness. Due to the short length of the lipid chains, the melting at the chain ends occurs gradually, increasing monotonically the area per lipid (APL)^73^ thus making the interfacial region less constraint, as detected by the az12 probe. As the temperature passes *T*_ph_, all lipid chain crystals melt and further temperature increase does not affect the area per lipid much, thus there is a drastic decrease of the d*S*_N3_/d*T* slope (Figure 3).

An APL increase with temperature can also cause enhanced penetration of water molecules to the interfacial region.^74,75^ However, the central frequency of az12 was found to be essentially independent of temperature, suggesting that reduction in steric hindrance (reduction of rigidity) rather than an increase in polarity with temperature, is reported by az12 in the interfacial region.

To better characterize the bilayer environments in the interfacial (az12) and hydrophobic (az11CN) regions we performed time-resolved 2DIR spectral diffusion experiments and measured characteristic times of their structural fluctuations and the extent of these fluctuations, characterizing their fluidities.

### 3.2 2DIR Spectral Diffusion Measurements

Figure 4 shows absorptive 2DIR spectra of the N_3_ diagonal peaks for az12 (top row) and az11CN (bottom row) in three bilayers at the waiting time of 0.1 ps and room temperature. Note that the blue peak is due to the 0 → 1 vibrational transition, while the red peak represents the 1 → 2 transition. The red bar indicates the center line for the 0 → 1 peak, connecting the local minima of slices parallel to the detection frequency axis (ω_*t*_). The slope of the central line at small waiting times reflects the amount of inhomogeneity for the transition. At small waiting times, *T*_w_, the 2DIR spectra are diagonally elongated due to a strong correlation between the excited and detected frequencies within inhomogeneous subensembles of the transition. As *T*_w_ increases, this correlation diminishes due to frequency fluctuations, leading to a rounder peak. This loss of frequency correlation, known as spectral diffusion, reflects the structural dynamics of the local environment of the reporter, the N_3_ moiety (Figure 5). Spectral diffusion can be quantified using the frequency–frequency correlation function (FFCF) by plotting the inverse of the center line slope (ICLS) as a function of *T*_w_.^76,77^ The FFCF represents the time-dependent probability that each frequency component within an inhomogeneous distribution will retain its original frequency. The decay of the ICLS with *T*_w_ reports on the normalized FFCF. By fitting the ICLS decay curve and using the absorption spectrum of the reporter, a complete set of FFCF parameters can be obtained.^76,77^ The normalized FFCF can be expressed as

2 $$C\left(t\right)=\left\langle \delta \omega \left(t\right)\delta \omega \left(0\right)\right\rangle =\delta \left(t\right)/T_{2}+\Sigma_{i}{\Delta_{i}}^{2}\exp\left(-t/\tau_{i}\right)$$

where δω­(*t*) is the shift in the vibrational frequency as a function of time, δ­(*t*) is the Dirac delta function and *T*_2_ is the homogeneous dephasing time. The homogeneous line width, Γ, can be derived from *T*_2_ as Γ = (π*T*_2_)^−1^. The initial value of the ICLS at *T*_w_ = 0 reports on the ratio of the homogeneous and inhomogeneous contributions to the peak. For each *i*th inhomogeneous contribution, Δ_*i*_ and τ_*i*_ indicate the line width of the frequency fluctuations and the associated spectral diffusion time constant, respectively. When τ_*i*_ is substantially slower than the experimentally accessible time window, a nondecaying component of the ICLS trace (a plateau) appears in the ICLS plot, often referred to as static inhomogeneity (Δ_0_), as it reflects the slow molecular motions that appear static relative to the experimental time window. The amplitudes, Δ_*i*_, obtained from the ICLS fitting are unitless parameters, which can be converted to wavenumbers (cm^–1^) by using the absorption spectrum as a reference (see ref 76 and Section S1 in the Supporting Information).

**Figure 4:**
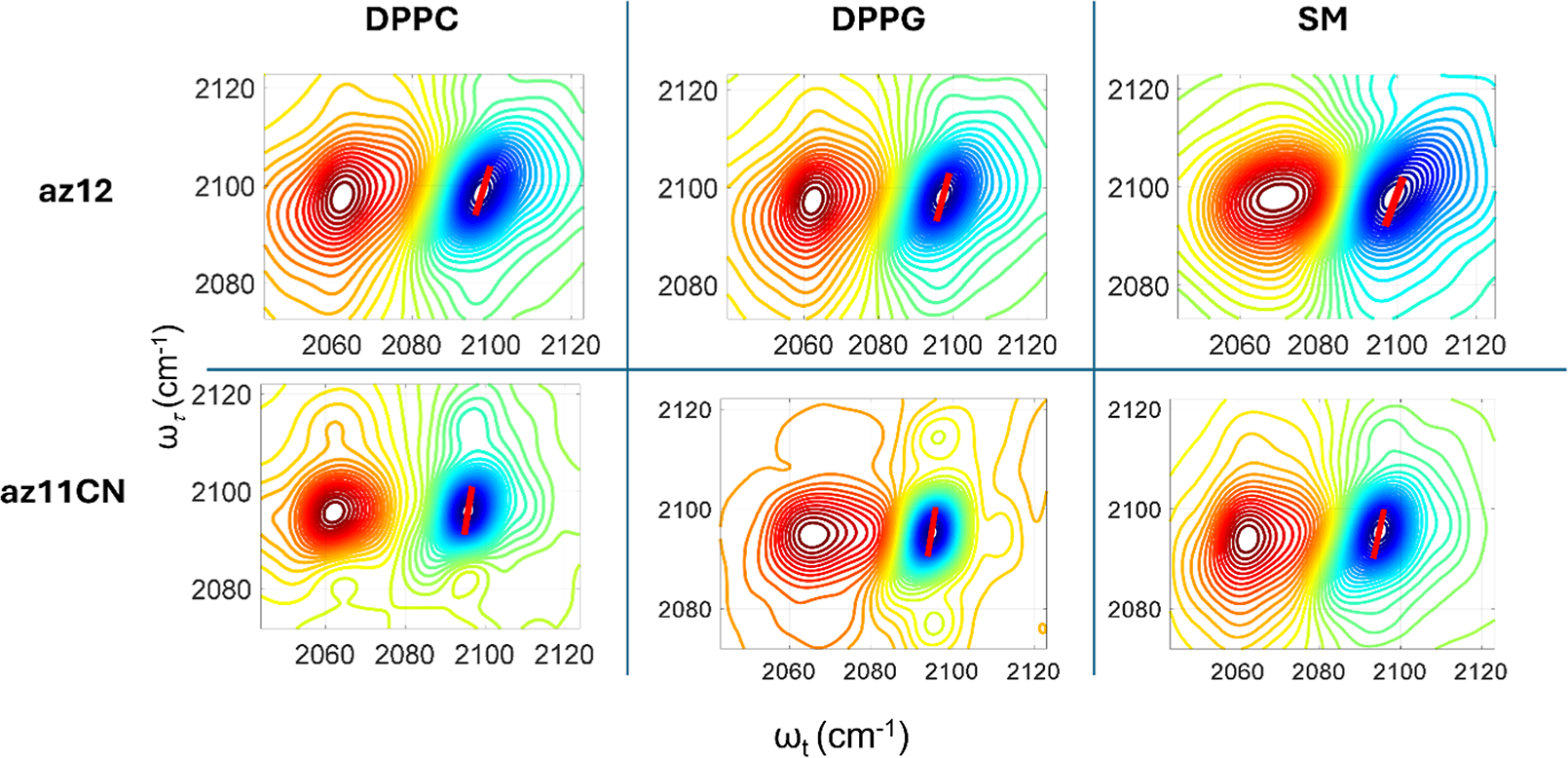
Absorptive 2DIR spectra of the az12 (top row) and az11CN (bottom row) probe compounds in the three bilayers measured at *T*_w_ = 0.1 ps at 25 °C.

**Figure 5:**
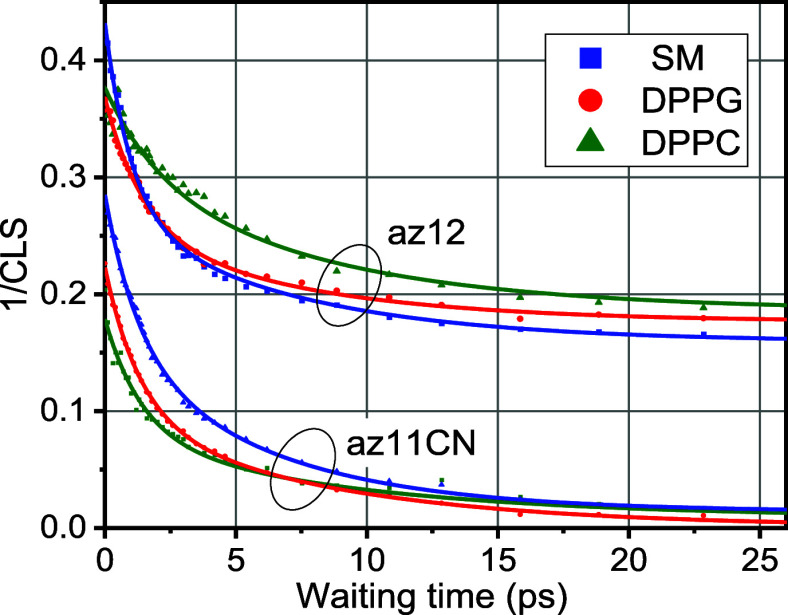
Waiting-time dependencies of the inverse center line slope of az12 and az11CN in the three bilayers indicated in the inset at 25 °C. Solid lines show fits with a biexponential function (see Table S1).

2DIR spectra of the probes in the bilayers were taken at three different temperatures: 25, 35, 45 °C. Figures 5, S2, and S3 display the ICLS decay curves for az12 and az11CN probes in the three bilayers at these temperatures, with the experimental data fitted to a biexponential decay function (fit parameters are shown in Table S1). The results reveal clear differences between the ICLS curves of az12 and az11CN. The decay of ICLS reflects the time scales for conformational equilibration in the environment, thus reporting on the fluidity of the environment. The fast and slow decay components for both probes are in the range of 1–2 ps and 5–9 ps; they are different for different bilayers, but the differences are not drastic (Table S1 and Figure 5). Other than the differences in decay constants, there are two major differences. First, the initial value of the ICLS is higher for az12 than for az11CN for all bilayers studied. Higher initial values of ICLS indicate larger inhomogeneity. This increased inhomogeneity for az12 arises from the higher polarity of the interfacial region relative to the hydrophobic core. Second, a distinct plateau is observed for az12 in all three bilayers. Such a plateau reflects conformations that do not equilibrate within the experimental time window (25 ps), indicating the presence of inhomogeneity that appears static, which is addressed in more detail later. Note that no such plateau was observed for az12 in solvents of polarities comparable to those of the interfacial regions (ethyl acetate) or higher (methanol).^50^

The fit of the ICLS traces with a double exponential function (eq 2) resulted in two inhomogeneous width (Δ_*i*_) and decay time (τ_*i*_) pairs, static inhomogeneous component (Δ_0_), and homogeneous line width (Γ) (see Table S1 and Section S1 for details). The decay times of ICLS (τ_1_, τ_2_) for az11CN are consistently decreasing at higher temperatures for all three bilayers (Table S1). Moreover, the decay time values for different bilayers are clustered at similar values at each temperature. For example, for az11CN, the fast decay component (τ_1_) averages ∼1.45, 1.1, and 0.6 ps at 25, 35, and 45 °C, respectively, in all three bilayers (Table S1). The slow component (τ_2_) is similar in different bilayers changing from ca. 8.5–7.5 ps and to 3.5 ps at 25, 35, and 45 °C, respectively. These observations indicate that the ultrafast dynamics in the hydrophobic region are largely insensitive to the lipid headgroup but strongly dependent on temperature.

The decay times for az12 are also similar for different bilayers at the same temperatures, taking for τ_1_ values of ca. 1.5, 1.2, and 0.9 ps for 25, 35, and 45 °C, respectively, and 7.0, 6.5, and 6 ps for τ_2_ for the same temperatures. Overall, the reduction of the decay times with temperature for az12 is not as severe as for az11CN, indicating the presence of stronger interactions in the interfacial region. Moreover, the characteristic times are not affected as strongly by the gel-to-liquid-crystal transition, compared to those for az11CN. Surprisingly, the az12 ICLS decay times for SM and DPPC/DPPG are not very different (Table S1), reporting on a similar bilayer fluidity in the tens of picoseconds window. Although hard to predict, larger differences could be found for other bilayer types.

Additional information on the rigidity of the environment can be derived from the inhomogeneous width data, Figure 6. The static inhomogeneous component (Δ_0_), homogeneous line width (Γ), and total inhomogeneous line width $\left(\Delta_{total}=\sqrt{\Delta_{0}^{2}+\Delta_{1}^{2}+\Delta_{2}^{2}}\right)$ are shown in Figure 6 for az12 (panel A) and az11CN (panel B) in the three bilayers at three temperatures. These parameters are linked mostly to the rigidity of the bilayer, although the homogeneous line width reports on the amplitude of ultrafast fluctuations, which can be linked to fluidity at an ultrafast time scale.

**Figure 6:**
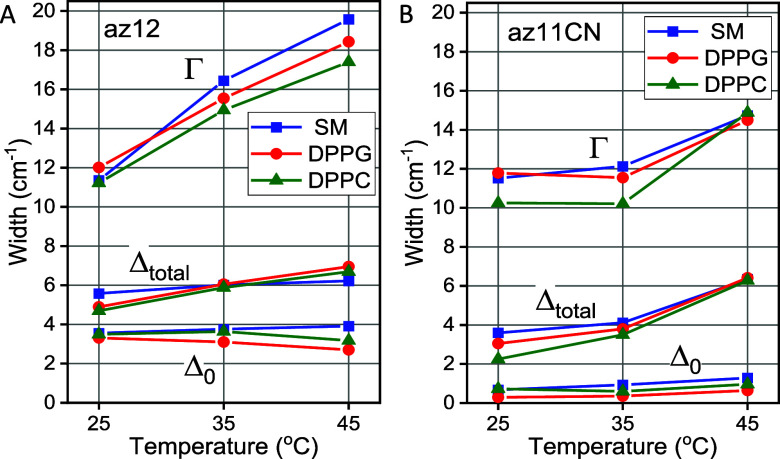
Static inhomogeneous component (Δ_0_), homogeneous line width (Γ), and total inhomogeneous line width $\left(\Delta_{total}=\sqrt{\Delta_{0}^{2}+\Delta_{1}^{2}+\Delta_{2}^{2}}\right)$ extracted from ICLS­(*T*_w_) fitting with eq 2 for az12 (A) and az11CN (B) in different bilayers at three temperatures.

The data for az11CN, which probes the hydrophobic region, are similar for all three bilayers (Figure 6B). The Δ_0_ contributions are very small, featuring significant errors. The total inhomogeneous and homogeneous widths increase with temperature, consistent with a phase transition occurring between 35 and 45 °C, where the increase is drastic compared to that in the 25–35 °C region. The increased inhomogeneity, Δ_total_, with temperature is associated with the reduction of the rigidity of the bilayer interiors, permitting larger width of θ distribution. An increase in Γ is associated with a decrease in the dephasing time (*T*_2_) of ν_N3_.

The az12 probe shows high values of static inhomogeneity, Δ_0_, for all three bilayers, which remains sizable at all temperatures, indicating that the interfacial region of the lipid bilayers is highly restrictive for the azido probe motion and that the restrictions are not much affected by temperature. These motion restrictions for the N_3_ moiety could be related to the N_3_ probe ability to form specific interactions in the interfacial region. In such case the reported dependences would be more specific to a particular binding site(s) rather than being more universal for the interfacial region in general. Such specific interactions are more likely to occur in SM. However, the similarity of the traces in Figure 6A for SM and the other two bilayers where specific interactions are much less likely suggests that specific interactions of the N_3_ moiety and the lipids are not strong enough to lead to a dominance of specific N_3_-lipid conformations. Weak affinity of the N_3_ moiety to hydrogen bonding facilitates such behavior of the probe. We conclude that the az12 probe constitutes a powerful reporter to measure rigidity (exerted angular constraints) and fluidity (time responses of the environment) in the interfacial region of a bilayer. Note that Δ_*i*_ and Γ parameters for az12 are affected by the higher polarity of the interfacial region so the direct comparison of these data with those for az11CN needs to take the polarity difference into account. Nonetheless, the enhanced static inhomogeneity in the interfacial region, not observed in the hydrophobic region, highlights distinct angular equilibration restrictions experienced by the azido group at the interface.

Satisfyingly, the az12 traces of Δ_0_, Δ_total_ and Γ for DPPC and DPPG are similar, essentially parallel with slight shifts (Figure 6A), echoing the behavior of their order parameters, *S*_N3_, and indicating similarities of the two interfacial regions despite the differences in their headgroups. An increase in Δ_total_ and a decrease in Δ_0_ with temperature is attributed to an increase in APL and softening of the interfacial regions, resulting in larger angular distributions and faster overall equilibration. An increase in Γ is caused by a decrease in the dephasing time, which may have a contribution from an increase of water permeability. Note, however, that no significant increase in polarity of the interfacial region was observed.

The az12 traces for SM differ from those for DPPC and DPPG showing a small growth of Δ_0_ and Δ_total_ and a significant increase in Γ. A larger increase of Γ for SM, compared to other two bilayers, may indicate a larger increase in water permeation to the interfacial region, which enhances fast fluctuations of the environment.^78–80^ Indeed, the reduction of the crystallinity with the temperature increase results in an increase in APL, providing space for enhanced water permeation. This conclusion agrees with the data highlighting the greater significance of hydrogen bonds in the interfacial region for SM relative to PG and PC.^81–84^

Consistent with prior studies, SM presents a stronger interfacial H-bonding network and more structured interfacial water than PC and PG bilayers, yielding higher interfacial order with weaker temperature sensitivity.^78–80,85^ The total inhomogeneity for SM is rather high already at 25 °C, in agreement with high polarity of its interfacial region, featuring both hydrogen bond donating and accepting groups,^70^ and potentially also associated with a larger amount of water molecules in the interfacial region.^38^ An increase of Δ_0_ and Δ_total_ with temperature for az12 in SM is very small, indicating the presence of stronger interactions in the interfacial region which prevent softening of the region at elevated temperatures. This observation resonates with the temperature behavior of the order parameter *S*_N3_ of az12, which shows the smallest change with temperature compared to DPPG and DPPC. The spectral diffusion data suggest that the mild decrease of the order parameter for az12 in SM at higher temperatures (Figure 3B) is mostly due to an increase of the homogeneous line width (Figure 6A), which can be linked to water permeation. On the contrary, the decrease of the order parameter at high temperatures for DPPG and DPPC is associated with an increase of the inhomogeneous line width and less with the water penetration into the interfacial region.

## 4 Conclusions

We have developed a vibrational probe, az12, capable of probing the rigidity, polarity, and ultrafast dynamics within the interfacial region of cell membranes using FTIR and 2DIR spectroscopies. Three lipid bilayers, DPPC, DPPG and SM, featuring differences in interfacial region and in the head groups, were interrogated with az12, targeting the interfacial region, and az11CN, targeting the hydrophobic region of a bilayer. We found that the az12 probe reports sensitively on the rigidity of the interfacial region and developed an order parameter, *S*_N3_, that is free from the polarity influence.

All three bilayers show phase transitions at respective temperatures manifested as an abrupt change of *S*_N3_ of az11CN, which is located in the hydrophobic region. Not surprisingly, no abrupt changes of *S*_N3_ of az12 were observed as its N_3_ moiety is located outside of the hydrophobic region that experiences the gel-to-liquid-crystal phase transition. Nevertheless, the interfacial region is strongly affected by change in temperature showing a monotonic decrease in rigidity with increasing temperature below *T*_ph_. At *T*_ph_, *S*_N3_ becomes essentially temperature independent, thus marking precisely the *T*_ph_ value. The monotonic decrease of the interfacial rigidity is explained as gradual softening of the bilayer in the gel phase, which ends at *T*_ph_ when the phase transition is completed. Thus, the two probes not only report precisely on the rigidity of the interfacial and hydrophobic regions but also shows how the rigidities of these two regions are correlated.

The 2DIR spectral diffusion data for az12 and az11CN probes resulted in a homogeneous line width (Γ) and several inhomogeneous line width contributions (Δ_0_ and Δ_total_) evaluated at different temperatures. The data revealed several key differences between the two probes, which include the presence of a large static inhomogeneous contribution for az12, absent for az11CN, and the presence of a phase transition for az11CN, absent for az12, manifested in a sharp increase of Δ_total_ across *T*_ph_.

The spectral diffusion data also reveal differences for different bilayers, most apparent for the interfacial region probed by az12. A greater increase in Γ for SM compared to that for DPPC and DPPG is caused by a larger decrease in the dephasing time, likely resulted from a larger increase in water permeability to the interfacial region of SM with temperature. Interestingly, the growth in Δ_0_ and Δ_total_ with temperature for SM is very small, indicating the presence of strong interactions in its interfacial region which prevent softening of the region at elevated temperatures, keeping the rigidity essentially constant at different temperatures. However, the rigidity of the interfacial region for DPPC and DPPG decreases significantly with temperature.

Both probes show other differences in the line width content for different bilayers. For example, the hydrophobic and interfacial regions of DPPC were found to be more rigid than those of DPPG, revealing the influence of the headgroup on the rigidity. Thus, the dynamics of both the interfacial region and the hydrophobic region are dependent on the headgroup type and on the interfacial lipid structure, offering a new simple approach for evaluating such rigidities. Interestingly, all the bilayer types feature a significant order at the interfacial region. Both probes show differences in spectral diffusion times for different bilayers, which report on the fluidity of the bilayer on the tens of picoseconds scale. Nonperturbative nature of the probes makes them attractive vehicles to assess rigidity and fluidity of any lipid bilayer.

Our work demonstrates that the angular restriction of the alkyl azido group can serve as a versatile probe in both polar and nonpolar environments, potentially beyond lipid bilayers. Furthermore, the use of complementary vibrational probes positioned in distinct regions of the bilayer provides a powerful strategy to address many open questions in membrane biophysics. For instance, one can now compare the effect of cholesterol in the hydrophobic and interfacial regions of a bilayer or ask a question whether unsaturation in the hydrophobic core has measurable effects on the interfacial region.

## Supplementary Material



## Notes

SI includes additional figures and details of 2DIR measurements, synthesis, sample preparation (PDF)
