# Supplementary material for: Probing Rigidity and Fluidity in the Interfacial Region of Lipid Bilayers with a Novel IR Probe
Source: J Phys Chem B. 2025 Oct 22;129(43):11220–9. doi: 10.1021/acs.jpcb.5c06183 (PMC12581132; doi:10.1021/acs.jpcb.5c06183)
Supplement: Supplementary file 1 [file jp5c06183_si_001.pdf]

Supplementary Materials

Probing Rigidity and Fluidity in the Interfacial Region of Lipid Bilayers with a Novel IR Probe

Md Muhaiminul Islam,* Sithara U. Nawagamuwage, Cameron A. Dennis, and Igor V. Rubtsov*

Department of Chemistry, Tulane University, New Orleans, Louisiana 70118, United States

Corresponding authors: mislam4@tulane.edu, irubtsov@tulane.edu

Table of contents

S1. Additional figures and data.

S2. Details of 2DIR spectroscopy measurements.

S3. Preparation of planar multilamellar bilayer samples.

S1. Additional figures and data

Temperature dependent FTIR spectra of az12 in DPPC, DPPG, SM and in two solvents, hexadecane and ethyl acetate are shown in Figurer S1.

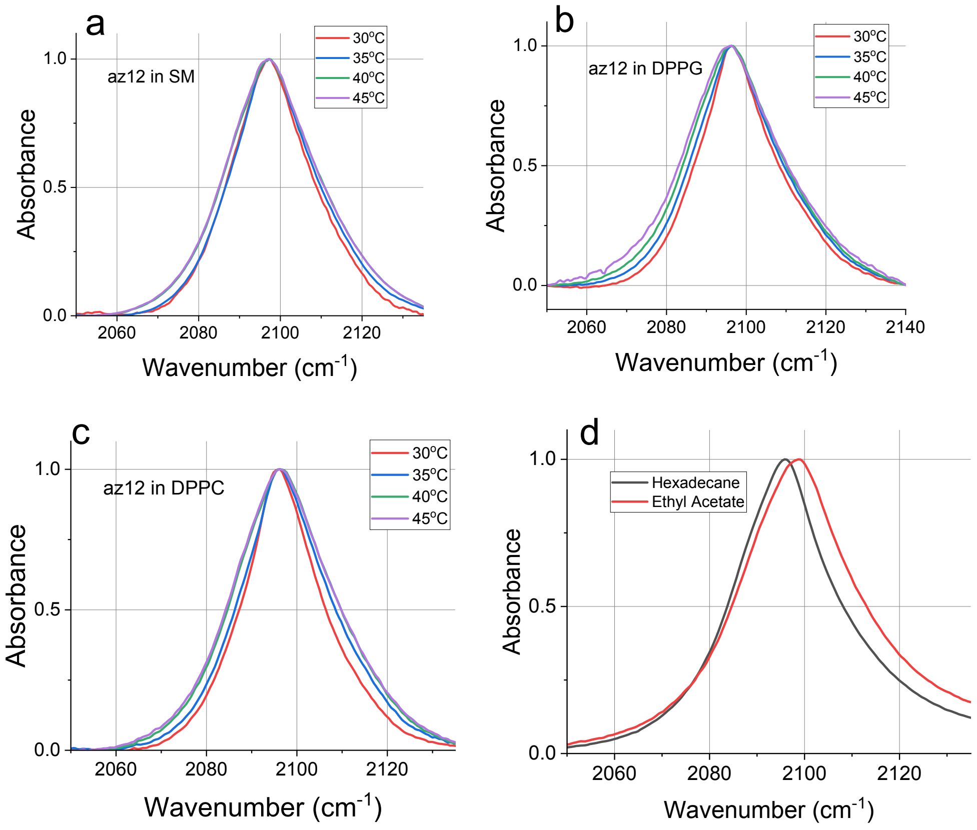

Fig. S1. Normalized, background-subtracted FTIR absorption spectra of ν_{N3} of az12 in SM (a), DPPG (b) and DPPC (c) bilayers at indicated temperatures and in two solvents indicated (d).

Waiting time dependences of the inverse center line slope of ν_{N3} for az12 and az11CN in SM, DPPC and DPPG at 35°C and 45°C are shown in Figures S2-S3.

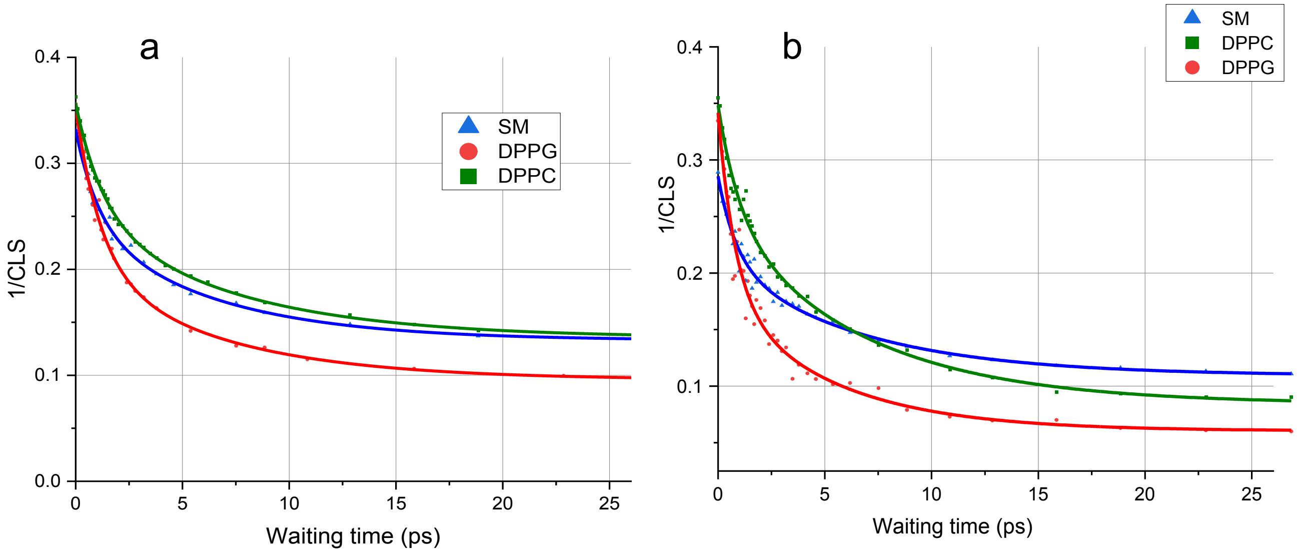

Fig. S2. Waiting time dependencies of the inverse center line slope of az12 in the three bilayers indicated in the inset at 35°C (a) and 45°C (b). Solid lines show fits with a biexponential function (see Table S1).

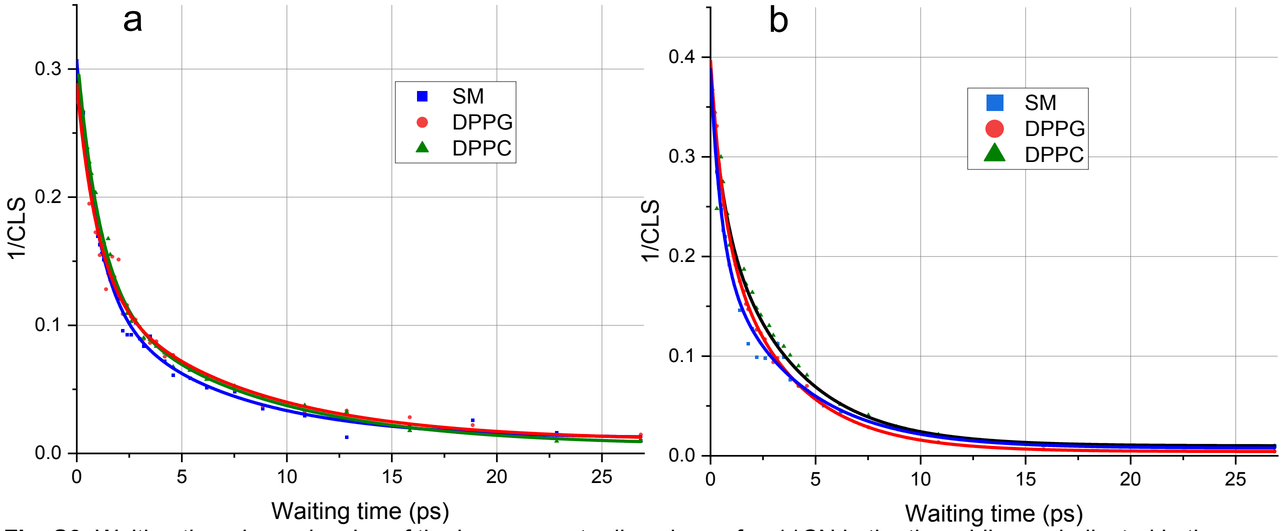

Fig. S3. Waiting time dependencies of the inverse center line slope of az11CN in the three bilayers indicated in the inset at 35°C (a) and 45°C (b). Solid lines show fits with a biexponential function (see Table S1).

Parameters of the FFCF obtained from the double-exponential fit of the 2DIR spectral diffusion data are shown in Table S1.

Table S1. FFCF parameters for az12 and az11CN in three lipid bilayers at three temperatures.

	Γ, cm^{-1}	Δ_{total}, cm^{-1}	Δ_0, cm^{-1}	τ_1, ps (Δ_1, cm^{-1})*	τ_2, ps (Δ_2, cm^{-1})*
az12, 25°C					
DPPC	11.2 ± 0.3	4.7 ±0.2	3.5 ± 0.1	2.0 ± 0.1 (2.0)	7.7 ± 0.2(2.4)
DPPG	12.0 ± 0.2	4.9 ± 0.2	3.3 ± 0.1	1.4 ± 0.1 (2.6)	7.2 ± 0.2(2.5)
SM	11.4 ± 0.2	5.6 ± 0.3	3.6 ± 0.1	1.3 ± 0.1 (3.5)	6.8 ± 0.3(2.5)
az12, 35°C					
DPPC	14.9 ± 0.4	5.9 ± 0.2	3.6 ± 0.1	1.3 ± 0.1 (3.1)	7.0 ± 0.2 (3.4)
DPPG	15.5 ± 0.4	6.1 ± 0.3	3.1 ± 0.1	1.2 ± 0.1 (3.9)	6.5 ± 0.3 (3.4)
SM	16.4 ± 0.3	6.0 ± 0.3	3.8 ± 0.1	1.1 ± 0.1 (3.0)	6.2 ± 0.2 (3.6)
az12, 45°C					
DPPC	17.4 ± 0.3	6.7 ± 0.4	3.2 ± 0.1	0.9 ± 0.1 (3.6)	6.5 ± 0.3 (4.7)
DPPG	18.4 ± 0.4	7.0 ± 0.3	2.7 ± 0.1	0.8 ± 0.1 (4.8)	5.2 ± 0.3 (4.3)
SM	19.6 ± 0.3	6.2 ± 0.3	3.9 ± 0.1	1.0 ± 0.1 (3.1)	6.0 ± 0.3 (3.7)
az11CN, 25°C					
DPPC	10.3 ± 0.3	2.3 ± 0.1	0.7 ± 0.1	1.4 ± 0.1 (1.7)	9.1 ± 0.3 (1.3)
DPPG	11.8 ± 0.4	3.1 ± 0.2	0.1 ± 0.1	1.3 ± 0.1 (2.0)	8.5 ± 0.3 (2.3)
SM	11.5 ± 0.3	3.6 ± 0.2	0.7 ± 0.1	1.5 ± 0.1 (2.6)	7.4 ± 0.2 (2.4)

az11CN, 35°C					
DPPC	10.2 ± 0.4	3.5 ± 0.1	0.6 ± 0.1	1.1 ± 0.1 (2.7)	7.4 ± 0.3 (2.2)
DPPG	11.5 ± 0.2	3.8 ± 0.1	0.4 ± 0.1	1.0 ± 0.1 (2.8)	7.1 ± 0.1 (2.5)
SM	12.1 ± 0.3	4.1 ± 0.1	0.9 ± 0.1	1.0 ± 0.1 (2.2)	6.1 ± 0.1 (2.2)
az11CN, 45°C					
DPPC	14.9 ± 0.3	6.3 ± 0.1	0.9 ± 0.1	0.6 ± 0.1 (3.5)	3.5 ± 0.3 (5.1)
DPPG	14.5 ± 0.5	6.4 ± 0.2	0.6 ± 0.1	0.6 ± 0.1 (4.0)	3.3 ± 0.2 (5.0)
SM	14.7 ± 0.4	6.4 ± 0.1	0.9 ± 0.1	0.5 ± 0.1 (4.4)	3.8 ± 0.3 (4.5)

* - inhomogeneity values, Δ_0, Δ_1 and Δ_2, were computed by eq. S1 and using the fit amplitudes a_0, a_1, a_2.

The linewidth parameters, Δ_i and Γ were determined by equations S1 and S2, following the methodology outlined by Fayer et al.[1]

$$\Delta_i = \sqrt{a_i}\, FWHM/(2\sqrt{2ln2}) \tag{S1}$$

$$\Gamma = b \times FWHM \tag{S2}$$

Here *FWHM* is the full width at half maximum of the ν_{N3} peak, a_i with i = 0, 1, 2, are the fit amplitudes of ICLS, and $b = 1 - \sum_i a_i$.

S2. Details of 2DIR spectroscopy measurements

The instrument and methodology for a fully automated dual-frequency three-pulse photon echo 2D infrared (2DIR) spectrometer with heterodyned detection have been described in detail previously.[2, 3] Briefly, the system utilizes an 800 nm laser operating at a 1 kHz repetition rate (Libra, Coherent) to pump two optical parametric amplifiers (OPA, Palitra, Quantronix) followed by two difference-frequency generation (DFG) units. The instrument incorporates advanced features, including beam direction stabilization accurate to 50 μrad, closed-loop phase stabilization accurate to 70 as, phase cycling, and automatic tuning of the phase-matching beam geometry. The three mid-IR pulses, each with pulse energies of approximately 1 μJ and parallel polarizations, were focused to a beam diameter of ~100 μm in the sample cell. Absorptive 2DIR spectra were obtained by summing the real parts of the rephasing and nonrephasing 2DIR spectra, individually phased using the respective pump-probe spectra. Center line slopes (CLS) were determined from the line connecting peak positions of one-dimensional cuts parallel to the pump axis at the ground-state bleach/stimulated emission (GSB/SE) peaks. Notably, the bending-libration combination band of water, observed near 2150 cm^{-1} and close to the azido-group peak, did not appear in the 2DIR spectra of MLBL due to its small extinction coefficient and broad linewidth.

S3. Preparation of planar multilamellar bilayer samples

Planar multilamellar bilayer (MLBL) samples were prepared using the Isopotential Spin-Dry Ultracentrifugation (ISDU) method, as adapted from Freed et al.[4-6] A test compound (az12 or az11CN) was mixed with a lipid (DPPC, DPPG, or egg SM, Avanti Polar Lipids) at a 1:10 molar ratio in chloroform. The mixture was dried under vacuum overnight to remove the solvent, followed by dissolution in water and sonication above the lipid's phase transition temperature to form unilamellar vesicles.

To assemble MLBLs, the unilamellar vesicle suspension was placed above a CaF_2 window (12 mm diameter, 1 mm thick) and subjected to ultracentrifugation at ~40,000 g for 18 hours at 20°C. This process facilitated gradual water evaporation, resulting in a highly aligned and optically clear MLBL with approximately two water molecules per lipid. The sample was subsequently hydrated with ~1 μL of H_2O,

sandwiched between two CaF_2 windows, and sealed in a custom-built cell holder. The water content, monitored via FTIR spectroscopy, remained stable at 13–15 water molecules per lipid; no significant changes in the bilayer properties were observed when water content exceeded 10 water molecules per lipid.

For precise temperature control during FTIR and 2DIR measurements, the sample cell was enclosed in an insulating jacket equipped with a temperature controller, maintaining temperature stability of ±0.2°C. Temperature was continuously monitored using a thermocouple attached to the CaF_2 substrate.

References

(1) Kwak, K.; Park, S.; Finkelstein, I. J.; Fayer, M. D. Frequency-frequency correlation functions and apodization in two-dimensional infrared vibrational echo spectroscopy: A new approach. *The Journal of Chemical Physics* **2007**, *127* (12). DOI: 10.1063/1.2772269 (acccessed 10/21/2024).

(2) Leger, J. D.; Nyby, C. M.; Varner, C.; Tang, J.; Rubtsova, N. I.; Yue, Y.; Kireev, V. V.; Burtsev, V. D.; Qasim, L. N.; Rubtsov, G. I.; et al. Fully automated dual-frequency three-pulse-echo 2DIR spectrometer accessing spectral range from 800 to 4000 wavenumbers. *Review of Scientific Instruments* **2014**, *85* (8). DOI: 10.1063/1.4892480 (acccessed 12/2/2024).

(3) Nyby, C. M.; Leger, J. D.; Tang, J.; Varner, C.; Kireev, V. V.; Rubtsov, I. V. Mid-IR beam direction stabilization scheme for vibrational spectroscopy, including dual-frequency 2DIR. *Opt. Express* **2014**, *22* (6), 6801-6809. DOI: 10.1364/OE.22.006801.

(4) Ge, M.; Budil, D. E.; Freed, J. H. ESR studies of spin-labeled membranes aligned by isopotential spin-dry ultracentrifugation: lipid-protein interactions. *Biophysical Journal* **1994**, *67* (6), 2326-2344. DOI: https://doi.org/10.1016/S0006-3495(94)80719-2.

(5) Borbat, P. P.; Costa-Filho, A. J.; Earle, K. A.; Moscicki, J. K.; Freed, J. H. Electron Spin Resonance in Studies of Membranes and Proteins. *Science* **2001**, *291* (5502), 266-269. DOI: doi:10.1126/science.291.5502.266.

(6) Chiang, Y. W.; Costa-Filho, A. J.; Freed, J. H. Two-dimensional ELDOR in the study of model and biological membranes. *Applied Magnetic Resonance* **2007**, *31* (3), 375-386. DOI: 10.1007/BF03166591.
